# On the Mechanism of Respiration

**Published:** 1847-04-01

**Authors:** 


On the Mechanism of Respiration. By *Francis Sibson*, Esq. (From the Philosophical Transactions, Part 4, 1846.) 4to. pp. 40. Plates.

This is an elaborate Essay from the pen of an able observer, of whose former labours in the same field we have had much pleasure of speaking favourably on another occasion.* Mr. Sibson's object is to distribute the respiratory muscles in a somewhat different order, as regards their inspiratory and expiratory action, to that now adopted. To this end he has minutely examined the anatomy in a great variety of animals, as reptiles, birds, mammals (some, we regret to say, by means of vivisection), and by a comparison of these with that of man, offers a new explanation of the mechanism. We regret that, with our limited space, and deprived of the aid of Mr. Sibson's beautiful engravings and ingenious diagrams, we despair setting his conclusions fairly before our readers, and must be content to refer those who feel interested in the subject to the work itself, easily accessible as it is, in forming a portion of the Philosophical Transactions.

